# Low-energy amplitude-modulated radiofrequency electromagnetic fields as a systemic treatment for cancer: Review and proposed mechanisms of action

**DOI:** 10.3389/fmedt.2022.869155

**Published:** 2022-09-08

**Authors:** Jack A. Tuszynski, Frederico Costa

**Affiliations:** ^1^ Division of Experimental Oncology, Department of Oncology, Cross Cancer Institute, University of Alberta, Edmonton, AB, Canada; ^2^ Dipartimento di Ingegneria Meccanica e Aerospaziale, Politecnico di Torino, Turin, Italy; ^3^ Autem Therapeutics, Hanover, NH, United States; ^4^ Oncology Department, Hospital Sírio-Libanês, São Paulo, Brazil

**Keywords:** electromagnetic fields, radiofrequency, cancer therapy, microtubules, metabolism, mitochondria, hepatocellular carcinoma, Warburg effect

## Abstract

Exposure to Low-Energy Amplitude-Modulated Radiofrequency Electromagnetic Fields (LEAMRFEMF) represents a new treatment option for patients with advanced hepatocellular carcinoma (AHCC). We focus on two medical devices that modulate the amplitude of a 27.12 MHz carrier wave to generate envelope waves in the low Hz to kHz range. Each provides systemic exposure to LEAMRFEMF *via* an intrabuccal antenna. This technology differs from so-called Tumour Treating Fields because it uses different frequency ranges, uses electromagnetic rather than electric fields, and delivers energy systemically rather than locally. The AutemDev also deploys patient-specific frequencies. LEAMRFEMF devices use 100-fold less power than mobile phones and have no thermal effects on tissue. Tumour type-specific or patient-specific treatment frequencies can be derived by measuring haemodynamic changes induced by exposure to LEAMRFEMF. These specific frequencies inhibited growth of human cancer cell lines *in vitro* and in mouse xenograft models. In uncontrolled prospective clinical trials in patients with AHCC, minorities of patients experienced complete or partial tumour responses. Pooled comparisons showed enhanced overall survival in treated patients compared to historical controls. Mild transient somnolence was the only notable treatment-related adverse event. We hypothesize that intracellular oscillations of charged macromolecules and ion flows couple resonantly with LEAMRFEMF. This resonant coupling appears to disrupt cell division and subcellular trafficking of mitochondria. We provide an estimate of the contribution of the electromagnetic effects to the overall energy balance of an exposed cell by calculating the power delivered to the cell, and the energy dissipated through the cell due to EMF induction of ionic flows along microtubules. We then compare this with total cellular metabolic energy production and conclude that energy delivered by LEAMRFEMF may provide a beneficial shift in cancer cell metabolism away from aberrant glycolysis. Further clinical research may confirm that LEAMRFEMF has therapeutic value in AHCC.

## Introduction

Liver cancer remains the fourth most common cause of cancer death and the second most lethal cancer in terms of 5-year survival, despite recent approvals of new systemic therapies (1). Patients with late-stage disease often lack adequate liver function and therefore may not be suitable candidates for systemic therapies. Exposure to low-power electromagnetic fields (EMFs) with specific frequencies is an emerging treatment for patients with advanced hepatocellular carcinoma (HCC) and other cancers (2–4).

In this review, we focus on medical devices that deliver low-power EMFs with a carrier wave frequency of 27.12 MHz, amplitude-modulated at multiple tumour type-specific or personalized envelope wave frequencies in the 10 Hz–150 kHz range (2, 3). The identification of specific amplitude-modulation frequencies is based on the physiological effects of exposure on excitable cells (5). This enables haemodynamic changes to be used as biomarkers of potentially therapeutic frequencies for particular cancers or for individual patients (6–8). We review evidence on the efficacy and safety of amplitude-modulated radiofrequency electromagnetic field (LEAM RF EMF) exposure in patients with HCC using these devices. The power delivered is far too low to cause detectable heating of cells or tissues (9). Rather, the effects of LEAM RF EMF exposure rely on the resonant interactions of electromagnetic waves with subcellular structures of normal cells and cancer cells in the human body (2–4, 10).

Radio waves interact with both moving and stationary electrical charges in cells, including atomic ions and molecular ions. When the frequency of the radio waves matches the timescales characteristic of cellular processes, exposure to the EMF may influence these processes by inducing movements of ions, because of resonant coupling of the oscillations. Cells partition charged molecules across membranes, which produces electrical and chemical potential differences, similar to those in a battery (11). These potential differences provide the ion-motive forces that underlie nearly all physiological processes in cells (12, 13). Cells also contain highly charged macromolecular structures, including microtubules, which are involved in cellular electrical signal propagation in addition to their well-known role in cell division (14, 15). We discuss potential mechanisms of action of LEAM RF EMF exposure in patients with cancer, at cellular and subcellular levels. These include potential overlapping effects of both the carrier wave and the envelope wave on ion flows, microtubules, mitochondrial localization and function, and cancer cell metabolism.

## Medical devices

Two existing medical devices provide systemic exposure to low-power LEAM RF EMFs with a carrier wave frequency of 27.12 MHz: the P1 (TheraBionic GmbH, Ettlingen, Germany) and the AutEMdev, an investigational device in development (Autem Therapeutics, Hanover, NH, USA). These small battery-operated devices emit extremely low-power EMFs, each delivering less than 100 mW into a spoon-shaped stainless-steel antenna that is placed into the patient's mouth (8, 9, 16). The resulting whole body-specific absorption rate of 1.77 mW/kg lies far below international safety limits and is too low to cause detectable heating (9). The device power is about 1,000 times lower than that of a mobile phone and 100,000 times lower than that of thermal tumour ablation devices (16). Furthermore, the frequency ranges differ from those used in telecommunications, where exposure to EMFs has controversial and inconsistent potential effects on health (reportedly both positive and negative) (17–20).

The carrier wave frequency of 27.12 MHz is one of the frequency bands reserved internationally for medical devices. Both devices use sinusoidal amplitude modulation of the carrier wave to generate envelope waves, with frequencies ranging from 10 Hz to 20 kHz for the AutEMdev and 0.1 Hz to 150 kHz for the P1 (8, 9). These envelope waves are generated individually in sequence at very precise frequencies, specified to three decimal places (21). For the P1 device, the envelope wave frequencies were selected as tumour-specific frequencies based on biofeedback responses defined by the magnitude of increased amplitude and/or the number of beats with increased amplitude of the radial pulse (5, 16). For the AutEMdev, personalized frequencies are selected in real-time by simultaneous monitoring of multiple haemodynamic parameters that may indicate potentially clinically beneficial effects in each individual (8).

Both the fixed 27.12 MHz carrier wave and the variable tumour-specific and/or patient-specific amplitude-modulated envelope waves may be involved in the physiological and therapeutic effects of EMF exposure. We hypothesize that electromagnetic waves at these frequencies couple and resonate with oscillations of charged macromolecular structures and ion flows within cells, similar to the way that a bell rings in sympathy when exposed to sound waves of the right frequency. This means that, although the device antenna is placed in the patient's mouth, the entire human body also becomes an antenna. With the exception of the bones, the effects of exposure to the carrier wave and the envelope waves are therefore distributed throughout the entire body.

In addition to the two above-mentioned devices, a third company (Novocure, Saint Helier, Jersey) has developed a treatment which exposes patients to so-called Tumor Treating Fields (TTFields) using the “Optune” device which is placed in the tumour region *via* an arrangement of two orthogonal sets of transducer arrays (22–24). These transducer arrays generate electric fields directed at the tumour site/cells and are activated sequentially on a second timescale. This enables a directional change of the incident electric field.

The intensity of TTFields is on the order of 1 V/cm and its optimal oscillation frequency depends on the tumour type. For example, glioblastoma multiforme patients are typically exposed to a frequency of 200 kHz. Therefore, TTFields involve frequencies which are two orders of magnitude lower than the carrier wave, and between two and five orders of magnitude higher than envelope frequencies used by LEAM RF EMFs. Also of note is that TTFields are entirely electric fields while LEAM RF EMFs are electromagnetic, i.e., they have both an electric and a magnetic component. Moreover, LEAM RF EMFs are applied systemically while TTFields are locally directed at the tumour site. Finally, LEAM RF EMF technology allows for a patient-specific frequency selection for the envelope wave while TTFields are tumour type-specific only.

**Figure 1:**
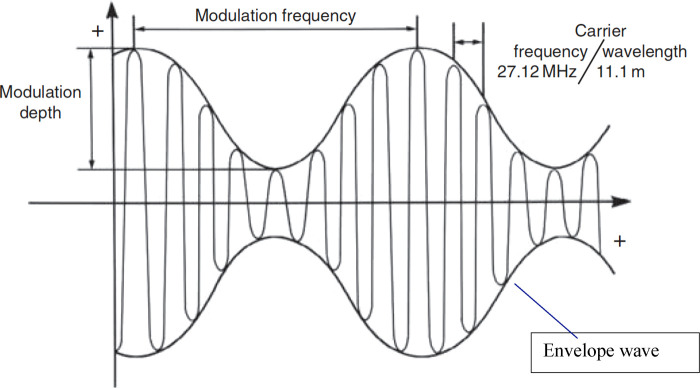
Schematic description of an amplitude-modulated radiofrequency electromagnetic wave. The carrier frequency (27.12 MHz) is sinusoidally modulated at specific frequencies (16).

The carrier wave is a high-frequency oscillation with amplitude modulated by a slow-frequency variation (Figure 1). This is a different mode of electromagnetic energy-based cancer therapy than TTFields. It also differs from therapies based on static and low-frequency magnetic fields and magnetic nanoparticles (25, 26). In Table 1 we have compared and contrasted the various wave-based (electromagnetic and ultrasound) technologies used in cancer therapy.

**Table 1:** Wave-therapy devices for oncology.

Medical devices	Energy	Operating frequency	Applicator	Tissue damage	Effect	HCC efficacy^a^
Radiofrequency ablation	Electric current	400 kHz	Needle	Heat	Local	Yes
Microwave ablation	Electric current	2,400 MHz	Needle	Heat	Local	Yes
High-intensity focused ultrasound	Ultrasound	0.2–3 MHz	MRI guided^b^	Heat	Local	No
Optune (Novocure)	Alternating electric current	100–300 kHz	Skin electrodes	Non-heat^c^	Local	Yes
AutEMdev and P1	Radiofrequency electromagnetic fields	Carrier (27 MHz) Modulation (0.1 Hz–150 kHz)	Spoon-shaped antenna	Non-heat^d^	Systemic	Yes

^a^ HCC, hepatocellular carcinoma.

^b^ MRI, magnetic resonance imaging.

^c^ Disruption of mitotic spindle in dividing cells.

^d^ Disruption of mitotic spindle in dividing cells, inhibition of ion flow *via* ion channels, destabilization of microtubules in G_0_ phase, and mobility inhibition of mitochondria, and therefore reduced cellular energy production.

## Physiological effects

A sleep-restoring effect in patients with insomnia was the first reported potential clinical benefit of LEAM RF EMF exposure, at specific effective envelope wave frequencies (27–29). A similar phenomenon was observed as mild, transient somnolence in patients with cancer undergoing LEAM RF EMF exposure, as described below. An effect of radiofrequency EMFs on the *α* waves of the human brain, which have a frequency of 8–13 Hz, may underlie these observations, and this may ultimately derive from modification of the behaviour of central nervous system neurons (30).

Alterations in haemodynamic regulation are also observed in patients exposed to LEAM RF EMFs. In studies using the P1 device, haemodynamic changes were detected as variations in skin electrical resistance, pulse amplitude and blood pressure. Frequencies eliciting the best biofeedback responses, defined by the magnitude of increased amplitude and/or the number of beats with increased amplitude of the radial pulse, were selected as tumour-specific (5). A study using the AutEMdev involved real-time automated monitoring of multiple haemodynamic parameters employing the AutEMdev and a separate monitoring device (6–8). Specific envelope wave frequencies that induced subtle but reproducible alterations in haemodynamic regulation were then identified in each individual. Similar effects on haemodynamic regulation have been reported for other EMF exposure modalities (31–33). These effects presumably result from modification of the behaviour of excitable cells in the heart and central/peripheral nervous system.

## Treatment of HCC

### *In vitro* and animal studies

A transverse electromagnetic cell culture system was used to mimic the effects of LEAM RF EMF exposure with devices using a 27.12 MHz carrier wave on cancer cell lines in two *in vitro* studies (9, 21). Growth inhibition of the Huh-7 and HepG2 liver cancer cell lines was greater with previously identified liver cancer-specific envelope wave frequencies than with breast cancer-specific frequencies or randomly selected frequencies (21). Conversely, growth inhibition of the MCF-7 breast cancer cell line was greater with breast cancer-specific frequencies than with liver cancer-specific frequencies (although this was not the case for the MCF-10A epithelial breast cell line) (21). Evidence for involvement of Ca^2+^ influx through the *CACNA1H* voltage-gated calcium channel in the anti-proliferative effects was provided by experiments in Huh7 and Hep3B cells (9). Ca^2+^ influx through this channel was only observed with liver cancer-specific frequencies, not with breast cancer-specific frequencies or randomly selected frequencies (9).

In mouse xenograft models of HCC, exposure to LEAM RF EMFs led to tumour shrinkage or reduced tumour growth, depending on the human tumour cell type injected (9). These effects were observed with liver cancer-specific envelope wave frequencies in the range of 100 Hz to 21 kHz, but not with randomly selected frequencies or sham exposure. Histological analysis indicated that tumour shrinkage was associated with differentiation of the injected cancer cells into quiescent cells with a fibroblast-like morphology (9). Similar findings have also been reported in xenograft models of metastatic breast cancer (34).

### Clinical studies

Five clinical study reports using the AutEMdev or P1 devices in patients with HCC have been published, to our knowledge. There is one study currently recruiting patients (Table 2). Barbault et al. described the selection of tumour type-specific envelope wave frequencies using the P1 device in patients with cancer, by monitoring variations in amplitude of the radial pulse (5). The range of these frequencies was higher than the frequencies previously identified in patients with insomnia (27–29), and also differed among patients with different cancers. Of the 1,524 frequencies identified across four cancer types, 170 were active in patients with HCC and 144 of these were specific to HCC. Anecdotal evidence of disease stabilization was provided in a small group of patients with cancer, of whom only one had HCC (5).

**Table 2:** Clinical studies of low-power AM RF EMF devices using a 27.12 MHz carrier wave in patients with HCC.

Reference	Patients	Device (envelope wave frequency range)	Regimen	Exploratory efficacy findings	Treatment-related adverse events
(35)	*N* = 38 with advanced HCC^a^	P1 (0.2 Hz–23 kHz)	40 min, thrice daily	OS: 20.7 weeks, ORR: 31.6%	Grade 1 mucositis, grade 1 fatigue
(5)	*N* = 163, 46 with HCC^a^ (frequency discovery) *N* = 28, 1 with HCC (treatment frequencies)	P1 (0.1 Hz–114 kHz)	60 min, thrice daily (self-administered)	Anecdotal	Grade 1 fatigue, grade 1 mucositis
(16)	*N* = 41 with advanced HCC^a^ and limited treatment options	P1 (100 Hz–21 kHz)	60 min, thrice daily (self-administered)	PFS: 4.4 months (95% CI, 2.1–5.3), OS: 6.7 months (95% CI, 3.0–10.2), PR: 9.8%	Grade 1 mucositis, grade 1 somnolence
(36)	*N* = 18 with advanced HCC^a^ (real-world study)	P1 (0.1 Hz–150 kHz)	60 min, thrice daily (self-administered)	OS: 6.7 months (95% CI, 4.4–9.5), PR 11.1%	Grade 1 anorexia, grade 1 mucositis, grade 1 GI bleed (baseline adjusted)
(6–8)	*N* = 66 with advanced HCC^a^	AutEMdev^b^ (10 Hz–20 kHz)	90 min, every 2–3 weeks (outpatient)	OS: 11.3 months (95% CI, 6.0–16.6)	Grade 1 somnolence
NCT04797884	*N* = 166 with advanced HCC (planned)	P1 (not disclosed)	60 min, thrice daily, every 6 weeks (self administered)^c^	Study recruiting	Study recruiting

AM RF EMF, amplitude-modulated radiofrequency electromagnetic field; GI, gastrointestinal; HCC, hepatocellular carcinoma; ORR, overall response rate; OS, median overall survival; PFS, median progression-free survival; PR, partial response.

^a^ Different patient characteristics make efficacy findings not directly comparable.

^b^ AutEMdev is an investigational device that is in development and has not yet received regulatory clearance or approval for commercialization.

^c^ Except first cycle as outpatient.

The HCC-specific frequencies identified by Barbault et al. were subsequently deployed in prospective phase 2 studies of the P1 device in patients with advanced HCC (Table 2) (5, 16, 35, 36). Although these studies were open-label and uncontrolled, evidence of antitumour effects was reported, with partial and complete radiological responses in small numbers of patients. In a pooled comparison, median overall survival for Child-Pugh B patients receiving TheraBionic P1 device as first line therapy was slightly higher than the 4.6 months median OS of historical controls receiving Sorafenib as first line therapy (36). These findings led to initiation of a multicentre, double-blind, randomized study comparing the P1 device with a placebo device in patients with advanced HCC following unsuccessful treatment with at least two lines of conventional systemic therapy (Table 2) (5, 16).

The AutEMdev deploys personalized frequencies in each patient (6–8). In an open-label clinical feasibility study in patients with advanced HCC (Table 2), personalized frequencies were identified in a 30-minute discovery phase using real-time multi-parameter haemodynamic monitoring. The selected frequencies were then applied in a subsequent 30-minute treatment phase. The subtle but reproductible haemodynamic alterations at personalized frequencies are hypothesized to represent a biological surrogate of antitumour activity (6–8). The studies using AutEMdev and P1 devices provided evidence for anti-tumour efficacy and/or maintained or improved health-related quality of life (HRQoL) in patients remaining on treatment (Table 2**)**, and overall survival in the prospective study cohort was longer compared with a retrospective control cohort (5–8, 16).

After hundreds of weekly procedures over periods of several years, mild, self-limiting somnolence or fatigue was the only frequently reported adverse event related to LEAM RF EMF exposure among patients receiving treatment with this modality alone or in combination with conventional anticancer treatment (Table 2). This side effect profile is consistent with the sleep-promoting effect of LEAM RF EMF exposure in patients with insomnia (27–29). The subtle effects of exposure on haemodynamic regulation do not appear to be associated with adverse events. Some patients receiving LEAM RF EMF exposure in combination with chemotherapy also experienced mild mucositis during exposure (5).

## Mechanisms of action

Carcinogenesis can be viewed as a phase transition in which normally synchronized, homogeneous, and differentiated cells in ordered tissues become asynchronized, heterogeneous, dedifferentiated, and proliferative cancer cells in disordered tumours (37). Resting membrane potentials are also altered (significantly lowered) and metabolism shifts from oxidative phosphorylation to glycolysis (11, 38–40). Because all subcellular processes occur at characteristic frequencies, these frequencies may also alter during carcinogenesis. This underlies the concept of favourable and unfavourable coherent frequency patterns in cancer, which may be manipulable using systemic LEAM RF EMFs at specific frequencies (4, 41). A similar phenomenon may also underlie the potential application of extremely low frequency EMFs to wound healing (42).

LEAM RF EMF exposure causes non-thermal effects in tissues (<1°C for nominal perfusion values). Experience with modelling of hyperthermal tumour damage indicates partial self-regeneration of normal tissue at tumour boundaries associated with perfusion by arterial blood should not be ignored (43, 44). Thermal damage can also change the interstitial structure and porosity of the tumour, according to recent mathematical models (45).

Although muscle cells and neurons were the first identified excitable cells (46, 47), nearly all cells have a resting membrane potential, and voltage-activated ion channels are expressed in non-excitable cells as well (48). One of these is the voltage-gated Ca^2+^ channel implicated in the anti-proliferative effects of LEAM RF EMF exposure in cancer cell lines *in vitro* (9, 21). Ion flows resulting from the motive forces provided by membrane potentials govern not only intracellular signalling and metabolism but also cell-to-cell communication (11).

Actin filaments, collagen, DNA, and microtubules are all highly charged linear macromolecular polymeric structures that can conduct electrical currents *via* the counterions that surround them (14, 49). This makes them potential candidates for resonant interactions with LEAM RF EMFs. Induction of DNA breaks has been proposed as a mechanism of action for potential anticancer effects of EMF exposure (42, 50–54). A direct effect of EMFs on mitochondrial electrochemistry has also been proposed (41). We consider, however, that microtubules are most likely the principal cellular bio-antenna for therapeutic LEAM RF EMFs due to their exceptionally high electric charge and dipole moment values directly coupling with EMFs. Below, we argue that the effect of exposure on microtubule ion flows may impair cell division and disrupt subcellular trafficking of mitochondria. We also hypothesize that LEAM RF EMF exposure alters cancer cell metabolism away from aberrant glycolysis, potentially inhibiting tumour growth.

### Microtubules

Microtubules are dynamic αβ-tubulin polymers that form part of the cytoskeleton and are involved in intracellular organization, organelle trafficking, and chromosome segregation. The cylindrical structure of microtubules is formed of 13 protofilaments of stacked tubulin dimers surrounding a 15 nm lumen, with a 25 nm outer diameter. Guanosine triphosphate-driven assembly and disassembly of tubulin dimers enables microtubules to exert push–pull forces, for example during cell division, which requires substantial force generation to segregate chromosomes. The adenosine triphosphate (ATP)-driven motor protein kinesin-1 tracks along microtubules, driving intracellular transport of mitochondria and other organelles.

Microtubules also support three established modes of ionic wave propagation: longitudinally on the surface, longitudinally within the lumen, and axially in and out of the nanopores between tubulin monomers (14). These ionic waves are formed by oscillation of counterions that are attracted to the charged inner and outer surfaces of the microtubule, and can be induced or modified by EMF exposure at the right frequency (49). Because microtubules are more conductive under specific ionic conditions than the cytoplasm, they act as the principal conduits for propagation of cellular ionic waves. Resonant coupling of EMFs with microtubules may therefore form the principal basis of the medical applications of EMFs (15). The specific frequency of oscillations depends on the length of the microtubule and the ionic strength and pH of the surrounding solution, and may also be influenced by cellular architecture (55, 56).

#### Carrier wave effects on microtubules

Microtubules are extremely longitudinally conductive to alternating currents at frequencies close to the 27.12 MHz carrier wave of the LEAM RF EMF medical devices, in the range of approximately 12–50 MHz (57–61). Furthermore, the highly charged C-terminal tail domains of tubulin protrude from the microtubule surface and also oscillate at frequencies close to that of the carrier wave (14). Although induced oscillations at these frequencies may disrupt microtubule function and motor protein traffic, the carrier wave frequency is constant and cannot therefore be responsible for the personalized or tumour-specific effects of specific envelope wave frequencies. Furthermore, an entire microtubule with a length of only 20 nm would resonate at 27 MHz, but most microtubules are much longer, spanning the length of the cell and achieving lengths up to 50 µm (or even higher under laboratory conditions). We therefore speculate that the carrier wave has a potentiating effect on ion flows along microtubules that renders cells susceptible to the effects of the lower-frequency envelope wave.

#### Envelope wave effects on microtubules

Spontaneous electrical oscillation of microtubules at fundamental resonant frequencies of 29 and 39 Hz has been reported in patch-clamp electrophysiological experiments using purified microtubule sheets and bundles, and permeabilized neurites *in vitro* (62, 63). Under constant holding potentials of 1 mV, microtubule-associated charges spontaneously oscillated at these frequencies, with a more than sixfold concomitant increase in conductivity (62, 63). These observations are consistent with axial ion flows in and out of the nanopores between tubulin monomers in a microtubule, and the frequencies fall within the range of envelope wave frequencies produced by LEAM RF EMF devices. Resonant frequencies of microtubules *in vivo* may differ from those observed in the *in vitro* electrophysiological studies because of differences in factors such as ionic strength, pH, protein-protein interactions, and cellular architecture. We therefore speculate that the combination of carrier wave and envelope wave interactions with different microtubule ion flows may underlie the physiological and potential clinical effects of LEAM RF EMF exposure.

#### Mitochondria

The subcellular positioning of mitochondria is tightly associated with their bioenergetic and cell-signalling functions. Mitochondria dynamically alter their subcellular localization by trafficking along microtubules using kinesin-1, and may also form a network enabling exchange of energy and signals between cells *via* membrane nanotubes (64–66). Perturbation of microtubules by EMF and resulting disruption of mitochondrial trafficking could therefore interfere with multiple mitochondrial functions. Consistent with this possibility, blocking mitochondrial anterograde trafficking by inhibiting integrin recycling led to detrimental amplification of reactive oxygen species in an *in vitro* study in breast cancer cell lines (67).

**Figure 2:**
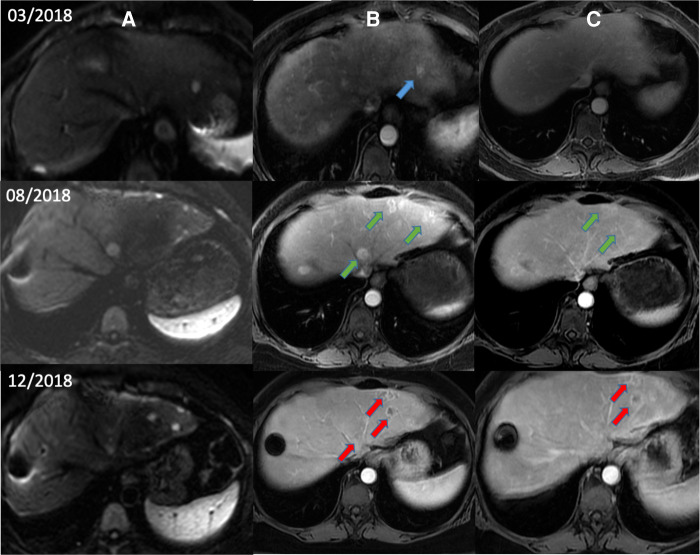
Magnetic resonance images from a patient with HCC after low-power amplitude-modulated radiofrequency electromagnetic field exposure. Diffusion-weighted imaging (**A**), arterial phase (**B**), and delayed phase (**C**) are shown. Patient with advanced HCC began treatment with sorafenib in March 2018 after diagnosis of recurrent HCC by diffusion and arterial phases (blue arrows). After documented radiological progression in August 2018 (green arrows), sorafenib was suspended and AutEMdev was started in 2-week intervals. In December 2018, lytic changes in the tumour lesions were observed by the arterial and delayed phases (red arrows). The patient was submitted to a surgical procedure to remove those lesions. Electronic micrographs of this patient are shown in Figure 3. HCC, hepatocellular carcinoma.

**Figure 3:**
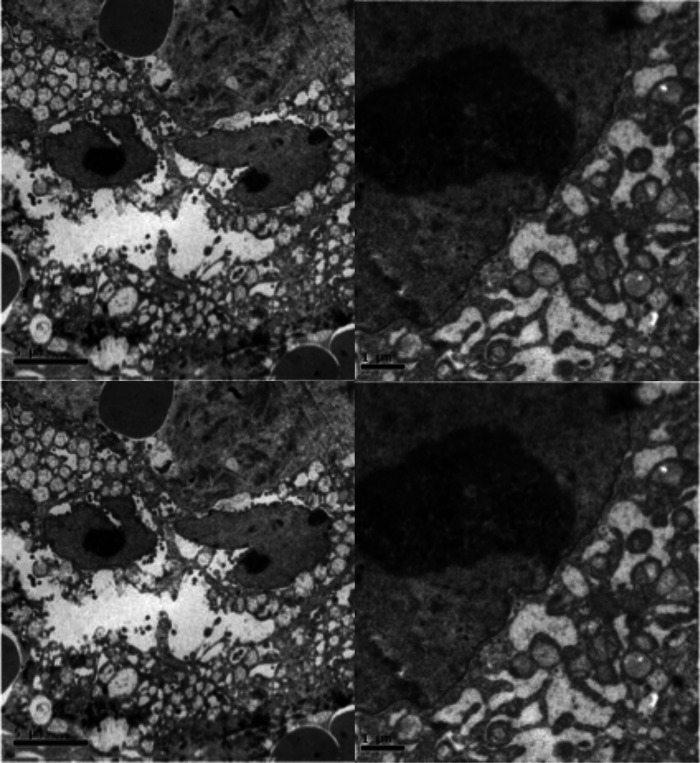
Abnormal mitochondrial morphology in a patient with HCC after low-power amplitude-modulated radiofrequency electromagnetic field exposure. Tissue sections at ultrastructural level of the patient given in Figure 2 are shown. Left: HCC cell with disorganized cytoplasmic organelles and diffuse electron-lucent dilation of reticulum, probably reflecting water accumulation, and mitochondria present in dilated regions. Right: HCC cell with hydropic dilated reticulum around isolated and atypical mitochondria showing morphological changes characterized by disorganized crista and swelling of the matrix. HCC, hepatocellular carcinoma.

In a hypothesis-generating and exploratory experiment, we used transmission electron microscopy to evaluate mitochondrial ultrastructure in liver tumour biopsies taken from patients with HCC who had undergone LEAM RF EMF exposure (Figures 2, 3). Mitochondria appeared to congregate in cellular locations distant from microtubules, and to show morphological changes characterized by disorganized crista and swelling of the matrix. To our knowledge, these abnormalities have not been previously reported in the context of HCC. This finding needs replication in future studies, but is consistent with effects of LEAM RF EMF exposure on mitochondria.

#### Metabolism

Malignant cancer cells produce lactate by fermentation of glucose even in non-hypoxic conditions, in a phenomenon known as the Warburg effect (38, 39). This is far less energy-efficient than oxidative phosphorylation for ATP production, but provides the cell with glycolytic intermediates to support biosynthesis of nucleotides and amino acids for cell growth (11). Because the Warburg effect is a defining feature of malignancy, reversing it has been proposed as a route to new cancer therapies, for example using microRNAs (40, 68, 69).

We hypothesize that LEAM RF EMF exposure may shift cellular metabolism away from glycolysis in cancer cells and hence lead to the restoration of the normal phenotype.(70) Although the effects of low-power AM RF EMF exposure are non-thermal, the energy delivered is ultimately dissipated as molecular motion. In the following sections, we calculate the power delivered to the cell and the energy dissipation due to EMF induction of ionic flows in microtubules, and compare this with total cellular metabolic energy production. We conclude that this energy delivery may contribute significantly towards shifting the cellular energy balance.

#### Estimation of power delivered to liver cells by EMF device

The starting point is the outcome of calculations is *in vitro* experiments with modulated radiofrequency using a previously published exposure system designed to replicate *in vivo* (human) conditions (16, 21). The sXcTEM027 exposure system apparatus (IT'IS Foundation, Zürich Switzerland) operating in the 27 MHz carrier wave region was used for *in vitro* experiments. The dosimetric assessments of 35 mm Petri dishes were performed for 2D culture cells in monolayer and suspension. The numerical dosimetry was verified by dosimetric temperature measurements. The non-uniformity of the specific absorption rate (SAR) distribution in the Petri dish filled with a small volume of cell culture media was determined and adjusted to represent SAR values determined in humans, as previously published (71). The heterogeneity of SAR with LEAM RF EMFs has not been evaluated in realistic tumour anatomies, to our knowledge, as has been performed for magnetic nanoparticle hyperthermia using micro computed tomography scans (72). Nevertheless, published dosimetry models for LEAM RF EMF provide an adequate starting point for estimating the power delivered to individual liver cells during exposure, as follows (16, 21, 71).

Dosimetric experiments with an RF EMF generator device were performed to estimate the specific absorption rate and distribution inside the human body. Numerical analysis based on the standardized anatomically based model of the human male (“Duke”) from the “Virtual Population” was performed and the simulation results were veriﬁed using SAR measurements in a simple rectangular phantom (73). Dosimetric experiments with radiofrequency in the MHz range exposure system for use during live cell imaging provided excellent exposure control and homogeneity.

Published dosimetry values for the P1 device calculate the average power delivered to a single human liver cell during exposure (9). Specific absorption rates were based on the “Duke” adult male model (weight 73 kg), one of ten models in the “Virtual Family” of whole-body anatomical models (73). The average power delivered by the EMF exposure device was 8.4 mW/kg for the liver (9). The average human liver weighs 1.3 kg and comprises 2.4 × 10^11^ cells (74), so we can readily calculate that the average power delivered per cell is approximately 50 fW. Thermal modelling was performed with a 2 mm voxel size in tissues. The thermal results reached a steady state after approximately 20–25 min with a maximum increase of 0.24°C for nominal perfusion values (75).

#### Estimation of EMF power dissipated by microtubules

The electrical current flowing through a single microtubule nanopore during oscillation at 29 Hz was estimated at 0.02–0.14 fA under an applied potential difference of 5 mV, in the electrophysiological study described above (62). From this, we can calculate the resistance of a single nanopore as 62.5 TΩ using Ohm's law (R = V/I). Ions flow in from one side of the microtubule wall and out from the other, so each pair of nanopores is a series resistor with a total resistance twice that of a single nanopore, equalling 125 TΩ. If a typical microtubule is 10 µm long and has 13 protofilaments each comprising 1,250 tubulin dimers, it contains 16,250 nanopores. Viewing this as a parallel arrangement of 8,125 nanopore pairs each with a resistance of 125 TΩ, the overall resistance of the microtubule can be estimated to be approximately 15 GΩ.

In a mitotic liver cancer cell, a microtubule plus-end is attached to a kinetochore and a minus-end interacts with a centrosome. If the potential difference across the microtubule in this setting is on the order of 1 mV, then we can calculate the current flowing as 0.07 pA from its resistance of 15 GΩ, again using Ohm's law. (47, 54–55) This translates into an ionic flow rate of 6 × 10^5^ unit charges per second per microtubule, which equates to 30 unit charges per second per nanopore for our typical microtubule with 16,250 nanopores. The frequency of charge transfer is therefore approximately 30 Hz. This is close to both the fundamental oscillation frequency in the electrophysiological study (62) and within the envelope wave frequency of the LEAM RF EMF devices. In this setting, one charge passes through each microtubule nanopore per cycle of the envelope wave, although the exact frequency would depend on cellular microtubule geometry and other factors. Nevertheless, the similarity in frequency between microtubule ion flows and the envelope wave provides a potential mechanism for transfer of electromagnetic energy into cancer cells.

We can also calculate the power dissipated by a single typical microtubule as 0.03 fW (using *P* = ½ V^2^/R since this represents the root mean square value of the power in an oscillating current situation described here, with the above values of 1 mV and 15 GΩ). Each kinetochore is attached to 30 microtubules and there are two copies of each of the 23 chromosomes, so we can estimate that there are 1,380 microtubules (excluding astral and polar microtubules) in a mitotic cell. The total power dissipated by microtubule ion flows in a single mitotic cell during oscillation at 29 Hz is therefore 46 fW, under the above assumptions. This value is very similar to the estimated power delivery of 50 fW per liver cell by the clinical EMF exposure device (see above).

#### Delivered EMF power relative to cellular metabolic power

We next consider what proportion of the normal cellular energy budget corresponds to the approximately 50 fW delivered by LEAM RF EMFs and dissipated by microtubule ion flows. We start by estimating the metabolic power of normal cells as 3 pW per cell using two separate methods. First, the human body uses about 100 W of metabolic power at rest and comprises 3.7 × 10^13^ cells on average, meaning that each cell uses 3 pW (76). Secondly, each cell contains about 2,000 mitochondria, which each produce about 30,000 ATP molecules per second by oxidative phosphorylation (74). ATP releases 30 kJ/mol of free energy upon hydrolysis or 5 × 10^–20^ J per molecule (76). Multiplying these values together also gives a metabolic power of 3 pW per cell. The estimates of approximately 50 fW for EMF power delivered and dissipated per cell therefore correspond to approximately 1.5% of metabolic power in normal cells.

In liver cancer cells, mitochondrial oxidative phosphorylation can be reduced by as much as 50%, depending on the disease stage, as cancer cells undergo the Warburg effect and shift to rely increasingly on glycolytic ATP production (77, 78). The human liver normally produces 30 mmol of ATP per minute by oxidative phosphorylation, or 3 × 10^20^ molecules of ATP per second, each releasing 5 × 10^–20^ J upon hydrolysis (35), which corresponds to a metabolic power of 15 W (or 15% of whole-body metabolic power).

Glycolysis is nine times less efficient than oxidative phosphorylation, so to maintain the same energy output, cells need to increase glucose consumption by an equivalent factor (77, 78). Consequently, 50 fW per cell may correspond to as much as 13.5% of glucose consumption in cells using glycolysis, compared with 1.5% in those using oxidative phosphorylation. Two-thirds of resting metabolic rate is devoted to heat production (76), so the energy delivered by EMFs may contribute meaningfully to the cellular energy budget. This may hence represent a significant shift in energy production away from glycolysis, which could counteract the Warburg effect and deprive cells of energy-rich metabolites produced from glycolysis (70) with a potential reduction in tumour growth and an overall decrease of the energetic burden the tumour imposes on the rest of the patient's body, consistently with the reported quality of life amelioration of the treated patients.

#### Summary of proposed metabolic hypothesis

We have shown that the frequency of charge transfer across isolated microtubules *in vitro* (approximately 30 Hz) falls within the envelope wave frequency range of therapeutic LEAM RF EMF devices. This frequency is likely to vary depending on cell morphology, physiology, and other factors. It may therefore fall within the same range as haemodynamically active and potentially therapeutic envelope wave frequencies (approximately 10–1,000 Hz). This provides a potential mechanism for transfer of EMF energy into cells, and dissipation of power *via* the ionic flows across microtubules. The power level is far too low to cause detectable heating of tissues, at about 8 mW for the whole liver and 50 fW per liver cell, but may nevertheless result in a significant shift of up to 13.5% in metabolic energy balance away from glycolysis. This could lead to suppression of the Warburg effect and promotion of a normal cellular metabolic phenotype. We hypothesize that this may be one of the mechanisms underlying the long-term disease control observed in patients with HCC in clinical trials. Direct effects of LEAM RF EMFs most likely involve subcellular electrically charged structures such as microtubules and possibly ionic flows around them, with predominant influence on mitosis. Hence, given a typically low mitotic fraction of cells within a tumour relative to the overall cell population and the much higher proportion of time cells reside within other phases of the cell cycle, a more complete explanation of long-lasting effects of EMFs on the tumour most likely requires metabolic effects which can endure long enough to affect cell fate/survival including a change of the phenotype mentioned above.

The structure of the cytoskeleton may underlie the potentially beneficial clinical effects of exposure to LEAM RM EMF using devices producing a 27.12 MHz carrier wave and envelope waves at specific frequencies in the range of approximately 10 Hz to 20 kHz. In particular, oscillating ionic flows may form in the condensed ions surrounding and within microtubules by resonant coupling with envelope waves of specific frequencies. These ionic flows may then lead to interference with ion channels, cell division, motor proteins, mitochondrial trafficking and morphology, and cellular energy balance. These putative mechanisms of action are potentially overlapping and not mutually exclusive, and all require further investigation by specifically designed assays. The efficacy of LEAM RF EMF in patients with HCC will be tested in randomized controlled studies. Nevertheless, we conclude that systemic exposure to LEAM RF EMFs of specific frequencies is a potential route to modifying the behaviour of cancer cells in patients with advanced disease.
